# Left or right? How social cues and task context influence relative width judgments and aperture choice

**DOI:** 10.3389/fpsyg.2026.1895232

**Published:** 2026-10-19

**Authors:** Ryosaku Makino, Hiroshi Matsui, Kentaro Kodama, Takayuki Tomono, Katsumi Watanabe

**Affiliations:** 1 Faculty of Sociology, Musashi University, Tokyo, Japan; 2 Graduate School, The University of Osaka, Osaka, Japan; 3 University Education Center, Tokyo Metropolitan University, Tokyo, Japan; 4 Department of Psychology, Sapporo Gakuin University, Sapporo, Japan; 5 Faculty of Science and Engineering, Waseda University, Tokyo, Japan

**Keywords:** action-oriented perception, human-like avatars, multiple apertures, passage preference, social cues, task context

## Abstract

People often need to choose a path through gaps formed not only by physical objects but also by other people. Although previous studies have shown that aperture perception and passing behavior are influenced by body-scaled physical constraints and social configurations, it remains unclear how such social information is used under different task contexts. The present study examined how the orientation and spatial configuration of three human-like avatars influenced judgments of two physically identical apertures. Two online experiments were conducted: a relative width-judgment task, in which participants chose the aperture that appeared wider, and a passage preference task, in which participants chose the aperture they would prefer to pass through. Generalized linear mixed models showed that the relative social structure of the two apertures, quantified as the difference in Degree of Preference (ΔDoP) between the right and left apertures, showed a similar association with choice across both experiments. In contrast, the effect of the center avatar direction (CAD) showed directionally opposite patterns across tasks: participants tended to choose the aperture aligned with the center avatar’s facing direction in the relative width-judgment task, whereas they avoided that direction in the passage preference task. A modest baseline tendency to choose the right aperture was observed across both experiments, and exploratory analyses indicated that the coupling between response time and confidence was stronger in the passage preference task. These findings suggest that social information is used differently across task contexts, with task dependence evident for the center avatar’s facing direction (CAD) but not for relative social configuration (ΔDoP).

## 1 Introduction

### 1.1 Aperture passing in physical and social environments

Perception of the environment is not determined solely by the physical properties of objects or spaces. From an ecological perspective, the environment is perceived in relation to the perceiver’s body properties and action possibilities (i.e., affordance: Gibson, 1979). For example, whether an aperture is passable depends not only on the physical dimensions of the environment, but also on the relation between those dimensions and the actor’s body scale (Warren and Whang, 1987). Studies on aperture passing have further shown that perceived passability and actual locomotor behavior are scaled to the body and action capabilities of the actor (e.g., Higuchi et al., 2011; Wagman and Taylor, 2005).

Most previous studies on aperture passing have examined perception and action toward a single aperture. However, in everyday environments, people often encounter multiple possible apertures and must choose one route from several alternatives. A limited number of studies have examined such multiple-aperture situations. For example, Shaw et al. (1995) investigated the selection of apertures in wheelchair locomotion, and Hackney et al. (2018) examined walking through multiple apertures arranged along a path. These studies suggest that aperture choice may depend not only on the relation between the perceiver and a gap, but also on the spatial relations among objects in the environment.

Additionally, in real-world environments, obstacles and apertures are not always composed of physical objects; they may also be formed by other people. Kendon (1990) described how people engaged in conversation organize a shared interactional space, or transactional segment, within an F-formation. An F-formation refers to a spatial arrangement in which individuals position and orient themselves to sustain shared social interaction, and passing through such a space may disrupt the ongoing activity. Thus, aperture passing in socially organized environments may be shaped not only by physical constraints but also by social-interactional constraints. Experimentally, Tomono et al. (2019) showed that the orientation of two people forming an aperture influenced participants’ passing behavior, particularly shoulder rotation angle and rotation direction. Similarly, Gregorj et al. (2023) showed that stronger social interaction within dyads increased avoidance by third parties and reduced the likelihood of pedestrians passing between group members. Together, these findings suggest that apertures formed by people are perceived and acted upon not solely in terms of physical width, but also in relation to the orientation and social interaction of the people forming them.

Thus, previous studies suggest that physical objects, social objects, and their spatial configurations can influence perception and action in aperture passing. However, it remains unclear how these influences change depending on the task context. In particular, the same socially structured aperture may be perceived differently when the observer judges its spatial properties and when the observer anticipates passing through it.

### 1.2 Task-dependent perception of socially structured apertures

The influence of socially structured apertures may depend on the task context in which they are perceived. The same spatial configuration may be evaluated differently when the observer judges its physical properties and when the observer anticipates acting within it. Thus, it is useful to distinguish between non-action-oriented perception and action-oriented perception. To examine this distinction, the present study employed two tasks: a relative width-judgment task, in which participants judged which aperture appeared wider, and a passage preference task, in which participants chose the aperture they would prefer to pass through.

The relative width-judgment task can be characterized as a third-person perceptual judgment, in which observers perceive the spatial properties of the environment without direct involvement in action. In such situations, the orientation of others is not intrinsically related to the physical structure of the aperture. However, previous research has shown that social cues such as gaze direction and head orientation can bias spatial attention (Frischen et al., 2007; Hietanen, 1999), suggesting that even perceptual judgments may be subtly influenced by socially relevant information.

In contrast, the passage preference task requires participants to anticipate their own movement through space and therefore reflects an action-oriented perceptual judgment. This type of task approximates a second-person perspective, in which the observer is implicitly involved in a potential interaction with others in the environment. Under such conditions, the orientation and configuration of others may function not merely as visual features but as socially meaningful constraints on action, such as indicating potential interference or interaction. Head and body orientation can also function as social-attentional cues, directing an observer’s attention toward the direction in which another person appears to be attending (Hietanen, 2002). From the perspective of personal space, people tend to maintain greater interpersonal distance in front of another person than behind them, and body orientation can influence judgments of interpersonal distance (Jung et al., 2016). Studies of mutual collision avoidance have shown that pedestrians anticipate others’ movements and mutually coordinate their behavior to avoid potential collisions (Honma et al., 2015; Murakami et al., 2022). Moreover, head orientation can provide predictive information about future locomotor direction and has been shown to contribute to predicting subsequent destination during locomotion in VR (Gandrud and Interrante, 2016). Thus, the facing direction of another person may provide action-relevant information for perceiving possible routes. In the present three-avatar configuration, the center avatar occupied a structurally distinct position; it was located directly in front of the observer and formed a shared boundary of both apertures. Accordingly, we hypothesized that its facing direction could influence aperture judgments in addition to the relative social configuration of the two apertures.

Based on this distinction, we hypothesized that the influence of social cues would differ across the two tasks. Specifically, width perception (Experiment 1) would primarily reflect the evaluation of spatial properties, whereas passage preference (Experiment 2) would incorporate socially structured constraints related to potential interaction. By comparing these two tasks, the present study aimed to clarify how social cues differentially shapes judgments under different task demands.

### 1.3 Objectives and hypotheses

The present study aimed to investigate how social cues and task context influence the perception and choice of apertures formed by human-like avatars (Figure 1). Specifically, we examined whether identical physical gaps would be perceived differently depending on the orientation and spatial configuration of the three avatars forming the two apertures. We hypothesized that socially structured spatial configurations would systematically influence both perceptual judgments and action-oriented judgments. We predicted that:

1. The relative social structure of the two apertures would influence relative width perception and choice behavior. In particular, differences in the spatial configuration of avatars were expected to modulate judgments even when the physical width of the apertures was identical.
2. The orientation of the central avatar, which provided directional information toward one of the two apertures, was expected to exert an additional influence on relative width perception and passage preference beyond the structural configuration of the apertures.
3. The influence of these social cues would differ depending on task context. Specifically, socially structured spatial information was expected to influence action-oriented judgments of passage preference (Experiment 2) differently from perceptual judgments of relative width (Experiment 1).

**Figure 1:**
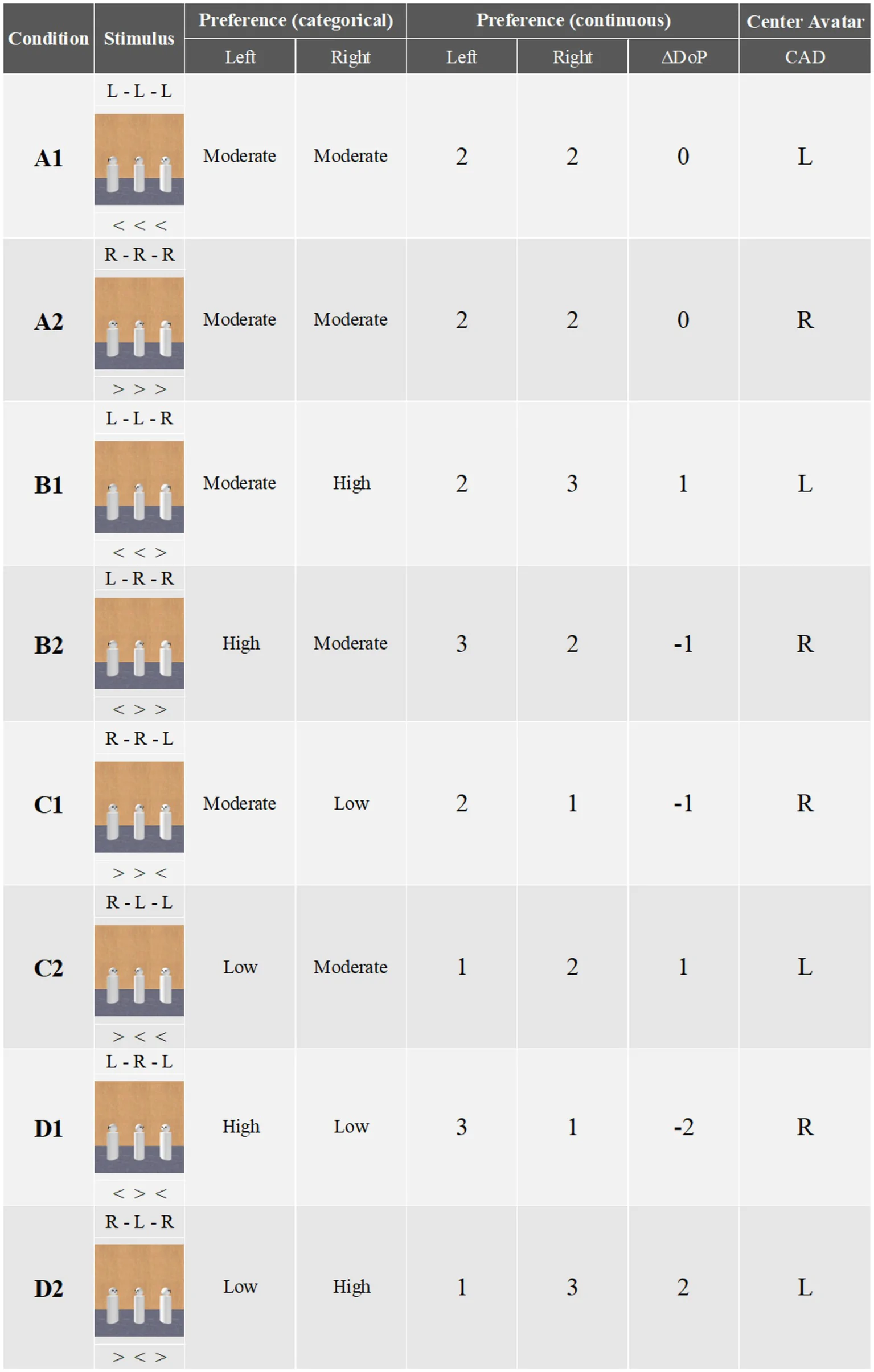
Experimental stimulus conditions used in both experiments. Each stimulus consisted of three avatars arranged at equal intervals, forming two apertures of identical physical width. Based on the orientations of adjacent avatars, apertures were categorized into three social configurations: face-to-face, back-to-back, and same-direction. Eight experimental conditions (A1–D2) were constructed and divided into two counterbalanced series, with Series 2 representing mirror reversals of Series 1. The relative structural difference between the two apertures was quantified using ΔDoP, ranging from −2 to +2, while CAD indicates the facing direction of the center avatar (L = left, R = right). DoP labels (Low, Moderate, High) represent the hypothesized ordering of the relative social configurations of the apertures based on avatar orientation.

To examine these hypotheses, we designed two online tasks using computer-displayed scenes: a relative width-judgment task (Experiment 1) and a passage preference task (Experiment 2).

## 2 Methods

### 2.1 Participants

Two experiments (Experiments 1 and 2) were conducted. We initially recruited 100 participants for each experiment through a crowdsourcing service (Yahoo! Crowdsourcing, Yahoo! JAPAN). Complete responses were obtained from 91 participants in Experiment 1 and 92 participants in Experiment 2, all of whom were included in the analyses. Nine participants in Experiment 1 and eight participants in Experiment 2 did not provide complete responses and were therefore not included in the analyses. No additional *post hoc* exclusion criteria were applied to the complete datasets. No *a priori* power analysis was conducted, and the final sample sizes were determined by the number of complete responses obtained. Demographic information regarding age, gender, and handedness was not collected. Participants were eligible if they were adults (18 years or older), were able to read Japanese, and voluntarily agreed to participate. No additional exclusion criteria were applied. This study was conducted in the Japanese language. A reward of 50 PayPay points (an electronic payment system available in Japan) was paid for each participant, which could be used for the service. Participants joined one of the two experiments, not both. Electronic informed consent was obtained from all participants prior to participation. This experiments were approved by the Ethics Review Committee on Research with Human Subjects at Waseda University and were conducted in accordance with the principles of the Declaration of Helsinki.

### 2.2 Apparatus and stimuli

The experimental stimuli and online task interface were developed using JavaScript-based software (p5.js, Vue.js, and Vuetify).

The visual stimuli consisted of eight experimental conditions (A1–D2), categorized into two Series (Series 1 and 2) to ensure a counterbalanced experimental design (Figure 1). Each stimulus consisted of three avatars arranged at equal intervals, forming two apertures of identical width. For each aperture, three distinct social configurations were possible based on the orientation of the two adjacent avatars: (1) “face-to-face,” in which the avatars face each other, suggesting potential social interaction, (2) “back-to-back,” in which the avatars face away from each other, and (3) “same-direction,” in which both avatars face the same direction.

In Series 1, four combinations of these configurations were presented as the left and right apertures (Conditions A1, B1, C1, and D1). Series 2 consisted of left–right mirror reversals of Series 1 (Conditions A2, B2, C2, and D2) to control for potential side biases. Thus, the stimulus set systematically varied the orientations of the three avatars while keeping the physical width of the two apertures identical.

To operationalize the relative social configuration of each aperture, we defined the Degree of Preference (DoP) as an *a priori*, hypothesis-driven index rather than a direct measure of participants’ subjective preference. Back-to-back, same-direction, and face-to-face configurations were assigned DoP values of 3, 2, and 1, respectively. This ordering was motivated by previous findings showing that interpersonal orientation and interaction structure influenced third-person avoidance and passage behavior (Kendon, 1990; Tomono et al., 2019; Gregorj et al., 2023). The numerical values of 1–3 represent an operational approximation of the hypothesized ordering rather than an empirically established interval scale.

For each stimulus, the relative configuration of the two apertures was represented by the difference in DoP (ΔDoP) between the right and left apertures, while the facing direction of the center avatar was represented as central avatar direction (CAD). This representation was used to distinguish the relative social configuration of the two candidate apertures from the directional social cue provided by the center avatar.

### 2.3 Experimental design

Both experiments used the same eight stimulus conditions shown in Figure 1. The stimuli were presented in random order. The two experiments differed only in task instructions. In Experiment 1, participants were asked to choose the aperture that appeared wider. In Experiment 2, participants were asked to choose the aperture they would prefer to pass through. Participants were recruited separately for the two experiments; thus, no participant took part in both tasks (between-subjects design).

### 2.4 Procedure

The procedure common to both experiments was as follows: After checking the consent form items on the computer screen, participants adjusted the size of the stimulus display using a standard credit card (85.6 × 54.0 mm) as a physical reference. They resized an on-screen rectangle until it matched the dimensions of their own credit card, thereby approximately standardizing the apparent stimulus size across different devices. Five practice trials were performed before the eight main trials. In the main experiment, each of the eight stimulus conditions was presented once, resulting in a total of eight trials per participant. The presentation order of the eight conditions was randomized for each participant. No separate attention-check trials were included. In each trial, participants were asked to gaze at a white cross on a black screen presented for 3 s. Subsequently, one of the eight conditions was presented for 5 s. The screen was switched to a screen where the participants were asked to choose either the left or right aperture using a button. Response time (RT) was recorded from the onset of the response screen, which appeared after the 5-s stimulus presentation, to the participant’s response. After each choice, participants also rated their confidence in the response on a 5-point scale. The experiment was completed in approximately 15 min.

### 2.5 Statistical modeling

To investigate the effects of social cues and task context on spatial width perception and passage preference judgment, we conducted a generalized linear mixed model (GLMM) analysis with a binomial distribution and a logit link function using the *lme4* package in R. In both Experiment 1 (relative width-judgment task) and Experiment 2 (passage preference task), the dependent variable was the participant’s choice (Choice, Left or Right).

The fixed effects included the difference in hypothesized Degree of Preference (DoP) between two gaps and the Center Avatar Direction (CAD, Left or Right). The DoP values are summarized in Figure 1. DoP was defined *a priori* based on avatar configuration, not estimated from participants’ choices or preference ratings. The DoPs were assigned to reflect the hypothesized strength of the spatial bias inherent in a gap consisting of two avatars. Specifically, the “Back-to-back” pair was predicted to exert the strongest directional bias (High DoP), the “Same-direction” pair was expected to yield moderate bias (Moderate DoP), and the “Face-to-face” pair was predicted to yield the minimum bias (Low DoP). For statistical modeling, CAD was effect-coded (left = −0.5, right = 0.5), allowing the model intercept to represent the average baseline tendency independent of avatar orientation.

In this study, we compared two types of models: one treating these DoP levels as categorical variables, and the other treating them as linear continuous variables. The difference in DoP (ΔDoP) is a continuous variable representing the structural bias inherent in the spatial configuration of the apertures. The DoP values were assigned as follows: the “Back-to-back” pair was scored as 3, the “Same-direction” pair as 2, and the “Face-to-face” pair as 1. ΔDoP was calculated by subtracting the score of the left gap from the score of the right gap (ΔDoP = DoP_Right_ − DoP_Left_), resulting in a centered variable ranging from −2 to +2. A positive value indicates a structural bias favoring the right gap, while zero indicates a balanced configuration.

ΔDoP and CAD were not orthogonally manipulated in the present stimulus set and were strongly associated (*r* = −0.816). We therefore assessed multicollinearity when both predictors were included in the same model. In the best-fitting combined GLMM, the variance inflation factors (VIFs) were 2.56 for ΔDoP and 2.54 for CAD. Given this association, the coefficients were interpreted cautiously as the effect of each predictor conditional on the other predictor being included in the model, rather than as evidence of experimentally independent effects.

To account for individual differences among participants, Participant ID (Sub) was included as a random effect (random intercept). While various model structures including interaction terms (e.g., ΔDoP × CAD) were tested, we present five representative candidate models that systematically test the influence of ΔDoP and CAD. CAD was effect-coded (left = −0.5, right = 0.5). Model selection was based on the Akaike Information Criterion (AIC).

Model 1: Choice ~ DoP level (categorical) + (1|Sub)

Model 2: Choice ~ ΔDoP (continuous) + (1|Sub)

Model 3: Choice ~ CAD + (1|Sub)

Model 4: Choice ~ DoP level (categorical) + CAD + (1|Sub)

Model 5: Choice ~ ΔDoP (continuous) + CAD + (1|Sub)

To directly examine whether the effects of the social cues differed between the two tasks (experiments), we additionally fitted combined GLMMs using data from both experiments. Experiment (task), ΔDoP, and CAD were included as fixed effects, with participant as a random intercept. Experiment and CAD were effect-coded (Experiment 1 = −0.5, Experiment 2 = 0.5; left-facing CAD = −0.5, right-facing CAD = 0.5). We compared an additive model, models including either the Experiment × ΔDoP or Experiment × CAD interaction, and a full model including both interactions. Model selection was based on AIC and likelihood-ratio tests.

Combined Model 1: Choice ~ Experiment + ΔDoP + CAD + (1|Sub_Exp)

Combined Model 2: Choice ~ Experiment × ΔDoP + CAD + (1|Sub_Exp)

Combined Model 3: Choice ~ Experiment × CAD + ΔDoP + (1|Sub_Exp)

Combined Model 4:Choice ~ Experiment × (ΔDoP + CAD) + (1|Sub_Exp)

## 3 Results

### 3.1 Descriptive statistics

Tables 1, 2 show the descriptive statistics for Experiment 1 (relative width-judgment task) and Experiment 2 (passage preference task), respectively. “Left” and “Right” indicate the number of trials in which each aperture was chosen, and “Right choice (%)” indicates the proportion of right-aperture choices for each condition.

**Table 1:** Descriptive statistics of aperture choices across the eight experimental conditions in Experiment 1 (relative width-judgment task).

Condition	Left (*n*)	Right (*n*)	Right choice (%)
A1	41	50	54.9
A2	30	61	67.0
B1	32	59	64.8
B2	41	50	54.9
C1	35	56	61.5
C2	30	61	67.0
D1	57	34	37.4
D2	22	69	75.8

**Table 2:** Descriptive statistics of aperture choices across the eight experimental conditions in Experiment 2 (passage preference task).

Condition	Left (*n*)	Right (*n*)	Right choice (%)
A1	21	71	77.2
A2	55	37	40.2
B1	10	82	89.1
B2	70	22	23.9
C1	71	21	22.8
C2	18	74	80.4
D1	78	14	15.2
D2	12	80	87.0

### 3.2 Experiment 1: Model comparison and selection

We compared five candidate models (Table 3). As a result, Model 1–5, which included both ΔDoP and CAD as fixed effects, yielded the lowest AIC (907.31), indicating that both stimulus configuration (i.e., the degree of preference, ΔDoP) and center avatar direction (CAD) were associated with relative width judgments after accounting for the other predictor in the model. We also tested a model including the interaction term (e.g., ΔDoP × CAD), but it resulted in a higher AIC and was therefore excluded.

**Table 3:** Comparison of candidate generalized linear mixed models for predicting participant choice.

Model	Fixed effects + random effect	AIC	ΔAIC
Model 1–1	DoP level (categorical) + (1 | Sub)	946.38	39.07
Model 1–2	ΔDoP (continuous) + (1 | Sub)	912.35	5.04
Model 1–3	CAD + (1 | Sub)	935.22	27.91
Model 1–4	DoP level + CAD + (1 | Sub)	938.45	31.14
Model 1–5	ΔDoP + CAD + (1 | Sub)	907.31	–

All models include random intercepts for participants (Sub). ΔAIC indicates the difference in Akaike Information Criterion relative to the best-fitting model. ΔDoP = difference in Degree of Preference; CAD = center avatar direction.

The best-fitting model (Model 1–5) revealed significant main effects for ΔDoP and CAD (Table 4). Specifically, the ΔDoP showed a significant positive effect (*β* = 0.66, SE = 0.12, *z* = 5.33, *p* < 0.001), indicating that apertures with higher preference scores (e.g., back-to-back configurations) were more likely to be selected as appearing wider.

**Table 4:** Summary of fixed effects for the best-fitting GLMM of Experiment 1.

Predictor	Estimate (*β*)	OR	SE	*z*-value	*p*-value	
(Intercept)	0.54	1.72	0.14	3.92	<0.001	^***^
ΔDoP	0.66	1.94	0.12	5.33	<0.001	^***^
CAD	0.78	2.18	0.29	2.65	0.008	^**^
Model fit metrics						
Marginal *R*^2^	6.4%					
Conditional *R*^2^	28.8%					

*β* = regression coefficient; OR = odds ratio; SE = standard error; *R*^2^ = coefficient of determination; ΔDoP = difference in Degree of Preference; CAD = center avatar direction. ^**^p < 0.01, ^***^p < 0.001.

Additionally, CAD had a significant positive effect (*β* = 0.78, SE = 0.29, *z* = 2.65, *p* = 0.008). When the center avatar faced right, participants were significantly more likely to choose the right aperture compared to when it faced left.

Furthermore, the model intercept was significantly positive (*β* = 0.54, SE = 0.14, *z* = 3.92, *p* < 0.001), indicating a baseline tendency to choose the right aperture. The random effect of participants indicated substantial individual variability (*σ*^2^ = 1.03).

In terms of odds ratios (OR), each one-unit increase in ΔDoP nearly doubled the odds of selecting the right aperture as appearing wider (OR = 1.94, 95% CI [1.52, 2.48]). Similarly, when the center avatar faced right, the odds of choosing the right aperture were approximately 2.18 times higher than when it faced left (OR = 2.18, 95% CI [1.22, 3.87]).

The final model (Model 1–5) yielded a marginal *R*^2^ of 0.064 and a conditional *R*^2^ of 0.288, indicating that the full model explained substantially more variance than the fixed effects alone.

### 3.3 Experiment 2: Model comparison and selection

We compared the same five candidate model structures in Experiment 1 (Table 5). Model 2–5, which included both ΔDoP and CAD as fixed effects, yielded the lowest AIC (724.70), indicating that both ΔDoP and CAD were associated with passage preference after accounting for the other predictor in the model. We also tested a model including the interaction term (ΔDoP × CAD), but it resulted in a higher AIC and was therefore excluded.

**Table 5:** Comparison of candidate generalized linear mixed models for predicting participant choice.

Model	Fixed effects + random effect	AIC	ΔAIC
Model 2–1	DoP level (categorical) + (1 | Sub)	1021.22	296.52
Model 2–2	ΔDoP (continuous) + (1 | Sub)	763.23	38.53
Model 2–3	CAD + (1 | Sub)	741.59	16.89
Model 2–4	DoP level + CAD + (1 | Sub)	742.46	17.76
Model 2–5	ΔDoP + CAD + (1 | Sub)	724.70	–

All models include random intercepts for participants (Sub). ΔAIC indicates the difference in Akaike Information Criterion relative to the best-fitting model. ΔDoP = difference in Degree of Preference; CAD = center avatar direction.

The best-fitting model (Model 2–5) revealed robust main effects for the ΔDoP and CAD (Table 6). Specifically, ΔDoP showed a significant positive effect (*β* = 0.60, SE = 0.14, *z* = 4.24, *p* < 0.001), confirming that apertures with higher preference scores were significantly more likely to be chosen for passage.

**Table 6:** Summary of fixed effects for the best-fitting GLMM of Experiment 2.

Predictor	Estimate (*β*)	OR	SE	*z*-value	*p*-value	
(Intercept)	0.31	1.36	0.13	2.37	0.018	^*^
ΔDoP	0.60	1.82	0.14	4.24	<0.001	^***^
CAD	−1.94	0.14	0.32	−6.03	<0.001	^***^
Model fit metrics						
Marginal *R*^2^	40.0%					
Conditional *R*^2^	50.2%					

*β* = regression coefficient; OR = odds ratio; SE = standard error; *R*^2^ = coefficient of determination; ΔDoP = difference in Degree of Preference; CAD = center avatar direction. ^*^p < 0.05, ^***^p < 0.001.

CAD had a highly significant negative effect (*β* = −1.94, SE = 0.32, *z* = −6.03, *p* < 0.001). In contrast to Experiment 1, when the center avatar faced right, participants were significantly less likely to choose the right aperture compared to when it faced left, indicating a strong tendency to avoid the direction of the avatar’s face.

Furthermore, the model intercept was significantly positive (*β* = 0.31, SE = 0.13, *z* = 2.37, *p* = 0.018), indicating a baseline bias toward choosing the right aperture. The random effect of participants showed moderate individual variability (*σ*^2^ = 0.67).

The odds ratio for ΔDoP was 1.82 (95% CI [1.38, 2.41]), indicating that spatial configuration remained a robust predictor of choice behavior. Notably, the CAD effect showed a much stronger, reversed impact compared to Experiment 1; when the center avatar faced right, the odds of choosing the right aperture dropped to only 14% of the odds observed when it faced left (OR = 0.14, 95% CI [0.08, 0.27]), reflecting a dramatic avoidance of the avatar’s face/gaze direction. The best-fitting model (Model 2–5) demonstrated a marginal *R*^2^ of 0.400 and a conditional *R*^2^ of 0.502, showing a substantial improvement in explained variance compared to Experiment 1.

### 3.4 Cross-experiment analysis

To directly test whether the effects of ΔDoP and CAD differed between tasks (experiments), we fitted combined GLMMs including Experiment and its interactions with these predictors. Adding the Experiment × CAD interaction significantly improved model fit compared with the additive model (*χ*^2^ = 106.87, *df* = 1, *p* < 0.001; AIC = 1628.85 vs. 1733.72). The Experiment × CAD interaction was significant (*β* = −2.68, SE = 0.27, *z* = −9.81, *p* < 0.001), indicating that the effect of CAD differed between the two tasks. In contrast, adding the Experiment × ΔDoP interaction to the model already containing the Experiment × CAD interaction did not improve model fit (*χ*^2^ = 0.05, *df* = 1, *p* = 0.829), and the Experiment × ΔDoP interaction was not significant (*β* = −0.04, SE = 0.19, *z* = −0.21, *p* = 0.837). Thus, the task-dependent difference was evident for CAD, whereas there was no evidence that the effect of ΔDoP differed between the two tasks (Table 7).

**Table 7:** Summary of fixed effects for the best-fitting combined GLMM.

Predictor	Estimate (*β*)	OR	SE	*z*-value	*p*-value	
(Intercept)	0.42	1.53	0.10	4.43	<0.001	^***^
Experiment	−0.21	0.81	0.19	−1.12	0.265	
ΔDoP	0.64	1.89	0.09	6.80	<0.001	^***^
CAD	−0.61	0.54	0.21	−2.85	0.004	^**^
Experiment × CAD	−2.68	0.07	0.27	−9.81	<0.001	^***^
Model fit metrics						
Marginal *R*^2^	27.2%					
Conditional *R*^2^	42.4%					

*β* = regression coefficient; OR = odds ratio; SE = standard error; *R*^2^ = coefficient of determination; ΔDoP = difference in Degree of Preference; CAD = center avatar direction. ^**^p < 0.01, ^***^p < 0.001.

### 3.5 Exploratory analyses of response time and confidence

Based on the GLMM results for the dependent variable Choice (3.2 and 3.3), and to further characterize the differences between Experiment 1 (perceptual judgment task) and Experiment 2 (action-oriented judgment task), we conducted exploratory analyses on post-stimulus response times (RT) and confidence ratings. RT represents the duration (in milliseconds) required for a participant to respond to the stimulus, while the degree of confidence in the response rated on a 5-point scale from 1 (lowest) to 5 (highest). Prior to analysis, RT data were trimmed to exclude trials with RTs shorter than 200 ms or longer than 4,000 ms from the onset of the response screen, following the previous study (Wolfe et al., 2010) to remove anticipations and attentional lapses. RTs were log-transformed to reduce positive skew before linear mixed-effects modeling.

#### 3.5.1 Model selection and effect of experiment on response time and confidence ratings

We evaluated the influence of task context, Experiment 1 (perceptual judgment task) and Experiment 2 (action-oriented judgment task), ΔDoP and CAD using linear mixed-effects models (LMM). For both dependent variables RT and Conf, model comparisons revealed that the simplest model including only Experiment as a fixed effect was sufficient to explain the data (RT: AIC = 919.56; Confidence: AIC = 2514.32). The addition of ΔDoP or CAD did not improve model fit for either RT (ΔDoP: AIC = 927.69; CAD: AIC = 925.79) or confidence (ΔDoP: AIC = 2521.17; CAD: AIC = 2520.78). More complex models including ΔDoP and CAD did not improve model fit, and neither ΔDoP nor CAD showed significant effects. These results indicate that the addition of ΔDoP or CAD provided little improvement in explaining RT and confidence ratings beyond task context.

Fixed-effects analysis of the selected models showed that Experiment was significantly associated with both RT and confidence ratings. Participants were significantly faster in Experiment 2 than in Experiment 1 (*β* = −0.08, *p* = 0.037). Furthermore, confidence was significantly higher in Experiment 2 (*β* = 0.40, *p* < 0.001). These results indicate that responses were faster and confidence ratings were higher in Experiment 2 than in Experiment 1.

#### 3.5.2 Correlations between response time and confidence ratings

Furthermore, to descriptively examine the relation between RT and confidence ratings in each experiment, we assessed the correlation between them using Spearman’s rank correlation coefficient. This non-parametric method was chosen to account for the ordinal nature of confidence ratings and the non-normal distribution of RT data. Because each participant contributed multiple trials, these correlations were treated as descriptive trial-level associations, and no inferential comparison of the correlation coefficients between experiments was performed.

Both experiments showed negative descriptive correlations between RT and confidence ratings, with a larger negative correlation observed in Experiment 2. In Experiment 1, the correlation was relatively weak (*ρ* = −0.117), indicating only a limited association between response time and subjective confidence. In contrast, Experiment 2 exhibited a larger negative correlation (*ρ* = −0.327). Descriptively, therefore, the RT–confidence association was stronger in Experiment 2 than in Experiment 1.

## 4 Discussion

### 4.1 Summary of findings

The comparative analysis of Experiments 1 and 2 yielded three main findings. First, both ΔDoP and CAD were associated with participants’ choices. Second, the combined analysis showed that the effect of CAD differed significantly between tasks, whereas there was no evidence that the effect of ΔDoP differed between tasks. Finally, exploratory analyses of RT and confidence provided additional information regarding differences between the two tasks.

### 4.2 Comparison of the best-fitting models

The present study demonstrated that both ΔDoP and CAD were associated with participant choice in both perceptual and action-oriented tasks. Importantly, the combined analysis provided a direct test of whether these effects differed between tasks. The Experiment × CAD interaction was significant, confirming that the effect of central-avatar direction differed between the two tasks. In contrast, the Experiment × ΔDoP interaction was not significant, providing no evidence that the effect of relative social configuration differed between tasks. Thus, the combined analysis supports a task-dependent modulation of the CAD effect, whereas there was no evidence of task-dependent modulation of the ΔDoP effect. This dissociation may reflect different functional roles of the two social cues: ΔDoP represents the relative social configuration of the two apertures and may therefore provide a comparatively stable relational constraint across tasks, whereas CAD provides directional information from the center avatar whose relevance may depend more strongly on whether participants judge relative width or anticipate passage. This finding suggests that configurational and directional social information may differ in their sensitivity to task context. The use of a GLMM allowed us to estimate trial-level choice probability while accounting for individual variability.

A notable difference between the two experiments was the substantially higher model fit observed in Experiment 2 compared to Experiment 1. Although direct comparison should be made cautiously because the two experiments were modeled separately, both marginal and conditional *R*^2^ values were higher in the action-oriented passage preference task, with the increase being particularly pronounced for conditional *R*^2^. This suggests that both fixed effects and individual variability contributed more strongly to explaining behavior under action-related conditions. One possible interpretation is that action-oriented tasks impose stronger constraints on the perception–action system, resulting in more stable and systematic behavioral tendencies. In Experiment 1, participants evaluated an ambiguous spatial property despite the physical widths being identical, which may have increased variability in responses. In contrast, anticipating movement in Experiment 2 may have organized perception relative to potential action, strengthening the coupling between environmental structure and behavior.

These findings extend previous studies on aperture perception and passing, which have primarily focused on possibilities in single-aperture situations (e.g., Warren and Whang, 1987; Higuchi et al., 2011; Tomono et al., 2019), by suggesting that relational structure among socially organized multiple apertures also systematically shapes behavior.

### 4.3 Similar influence of ΔDoP

Across both experiments, ΔDoP emerged as a robust and consistent predictor of choice. Regardless of whether participants were asked to judge which aperture appeared wider or which aperture they would prefer to pass through, larger positive values of ΔDoP systematically increased the probability of choosing the right aperture (Figures 2, 3). This consistency indicates that relative differences in socially structured configurations between the two gaps exerted a stable influence across task contexts. Importantly, ΔDoP captured the graded structural contrast between the two apertures rather than treating stimulus configurations as isolated categorical conditions. The fact that the continuous representation of ΔDoP provided a better model fit than categorical alternatives suggests that participants were sensitive to incremental differences in the relational social organization of the avatars. This finding is consistent with previous studies on multiple-aperture crossing, which have shown that environmental layout influences path selection and passing behavior beyond the absolute width of a single gap (Shaw et al., 1995; Hackney et al., 2018). However, whereas those studies mainly examined physically structured apertures and actual locomotor trajectories, the present study extends this perspective to socially structured apertures. Specifically, our findings suggest that relational social configuration can bias perceptual judgments and passage preference even when the physical width of the two apertures is identical.

**Figure 2:**
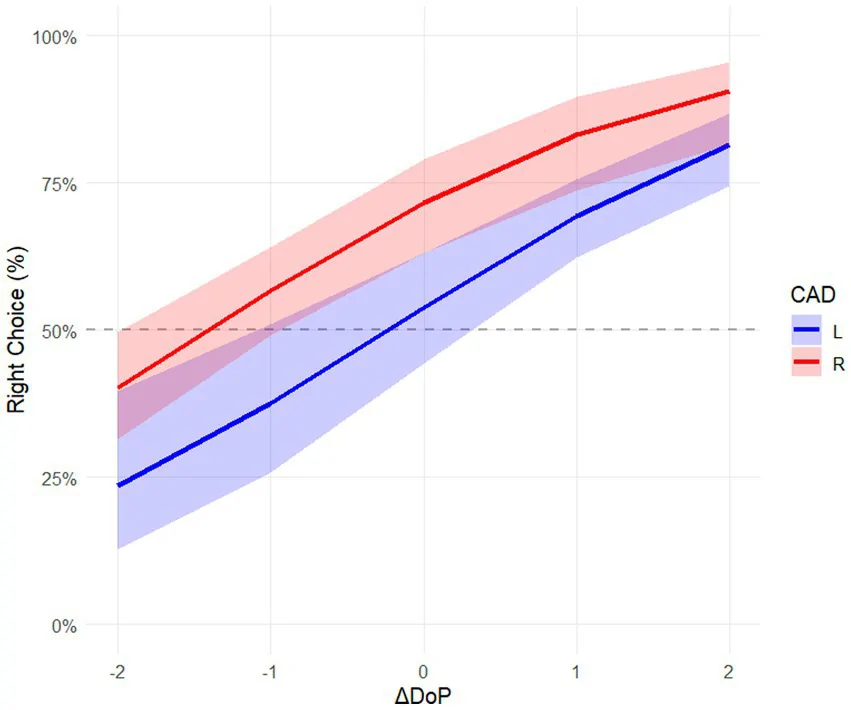
Right choice (%): predicted probabilities of choosing the right aperture in Experiment 1 (relative width-judgment task) based on the best-fitting additive model. The probability of choosing the right aperture increased systematically with ΔDoP. In addition, participants were more likely to choose the right aperture when the center avatar faced right (red line) than when it faced left (blue line). CAD indicates center avatar direction (L = left-facing, R = right-facing). Shaded areas indicate 95% confidence intervals.

**Figure 3:**
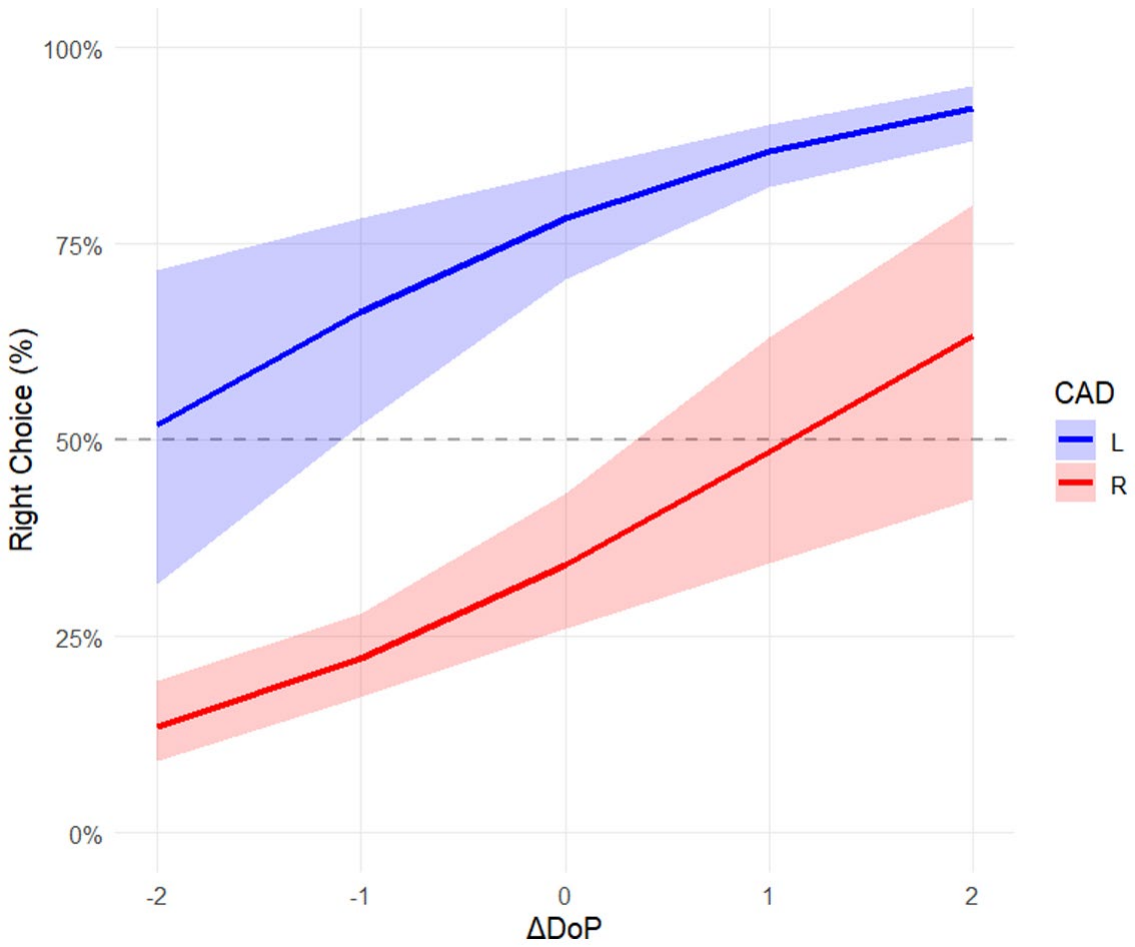
Right choice (%): predicted probabilities of choosing the right aperture in Experiment 2 (passage preference task) based on the best-fitting additive model. The probability of choosing the right aperture increased systematically with ΔDoP. In addition, participants were more likely to choose the right aperture when the center avatar faced left (blue line) than when it faced right (red line). CAD indicates center avatar direction (L = left-facing, R = right-facing). Shaded areas indicate 95% confidence intervals.

Although the physical width of the apertures was identical in the present study, the structural organization of avatar orientations introduced asymmetries in perceived spatial structure. ΔDoP therefore appears to function as an index of relative structural bias embedded in the socially organized layout of the environment. Overall, these results provide clear support for Hypothesis 1, demonstrating that socially structured spatial configurations systematically shape both perceptual and action-oriented judgments. One possible interpretation is that participants were sensitive to the relational organization of the avatars, rather than to isolated directional cues.

### 4.4 Reversal of CAD influence

The most striking finding of the present study was the opposite effect of CAD between the width perception and passage preference tasks. While participants in Experiment 1 showed a slight tendency to choose the aperture aligned with the center avatar’s facing direction, participants in Experiment 2 showed a strong tendency to avoid that direction. This reversal may suggest that the directional cue provided by the center avatar was interpreted differently depending on whether the task involved perceptual evaluation or anticipated action. This finding is consistent with the framework introduced in the Introduction, which proposed that socially structured apertures may be perceived differently depending on task context. In Experiment 1, the center avatar’s orientation may have functioned primarily as a spatial-attentional cue. Previous research has shown that gaze direction and head orientation automatically bias visual attention toward the indicated direction (Hietanen, 1999), and such effects might be closely related to joint attention mechanisms (Tomasello, 2009).

In contrast, when participants anticipated passing through one of the apertures in Experiment 2, the same directional cue appeared to acquire action-related meaning. From the perspective of social affordances, one possible interpretation is that the center avatar’s orientation may have specified not only where the avatar was facing, but also what possibilities for action it afforded the participant, such as potential interference, collision risk, or avoidance. This view is compatible with ecological accounts of social interaction, which emphasize that people perceive what others afford for their own action and coordination within a shared environment (Valenti and Gold, 1991; Marsh et al., 2009). These interpretations are consistent with empirical studies suggesting that body orientation and gaze direction provide information about another person’s future movement and influence navigation-related behavior (e.g., Nummenmaa et al., 2009; Tomono et al., 2019). In the present study, the center avatar, which occupied a privileged position between the two candidate apertures, may have been perceived not merely as part of the spatial layout but as a potential interaction partner or moving agent. As a result, participants may have avoided the aperture aligned with the avatar’s facing direction in order to avoid potential interference or collision.

This interpretation is also consistent with studies on interpersonal avoidance behavior, such as the “hesitant avoidance” phenomenon observed when two pedestrians approach each other head-on (Honma et al., 2015). Although the present study used static avatars and did not involve actual locomotion, the findings suggest that even static directional social cues can influence choice behavior when action is anticipated. At the same time, recent studies have suggested that real-world collision avoidance depends not only on static directional cues but also on dynamic mutual anticipation between pedestrians (Murakami et al., 2022). Future research using interactive or locomotor paradigms will therefore be necessary to examine how directional cues and mutual anticipation jointly shape behavior in socially structured environments. Taken together, these findings provide support for Hypotheses 2 and 3, suggesting that the influence of directional social cues may depend on task context and action relevance, although the specific mechanisms underlying this task-dependent effect remain to be determined.

### 4.5 Ad-hoc evidence from response time and confidence ratings

The exploratory analyses of RT and confidence ratings further supported the task-dependent differences in post-stimulus response characteristics. Across both experiments, RT and confidence ratings were negatively correlated, indicating that faster responses were accompanied by higher subjective certainty (Mamassian, 2016). However, this relationship was substantially stronger in the passage preference task (Experiment 2) than in the relative width-judgment task (Experiment 1). In Experiment 1, participants judged which aperture appeared wider despite the physical widths being identical, requiring evaluation of an ambiguous spatial structure with no objectively correct answer. This may have increased response variability and weakened the coupling between RT and confidence ratings. In contrast, Experiment 2 required participants to anticipate movement through one of the apertures, thereby organizing perception relative to potential action. Under such action-related constraints, RT and confidence ratings became more strongly coupled, suggesting that passage preference responses were accompanied by greater temporal stability and subjective certainty. These exploratory findings therefore characterize response behavior after stimulus viewing but do not directly test the mechanism underlying the CAD effect or provide an independent test of Hypothesis 3.

### 4.6 General rightward preference across tasks

An additional finding, not directly predicted by our hypotheses, was a modest baseline tendency to choose the right aperture across both experiments. After effect-coding CAD, the model intercepts remained significantly positive in both Experiment 1 and Experiment 2, suggesting that this tendency was not simply attributable to the direction of the center avatar or the structural configuration of the apertures. This result is broadly consistent with previous reports of rightward biases in spatial attention, navigation, and spatial expectations (e.g., Robertson et al., 2015; Zhao et al., 2025). Because this effect was exploratory and not central to the present hypotheses, it should be interpreted cautiously and examined further in future studies.

### 4.7 Limitations and future directions

Despite the theoretical implications of the present findings, several limitations should be acknowledged. First, the study was conducted as an online web-based experiment in which participants selected between apertures using button responses. Although this design enabled controlled manipulation of avatar configurations, it did not involve actual locomotion or real-time interaction. Accordingly, the findings reflect anticipatory perceptual judgments rather than fully realized behavior in physical environments. Participants’ body dimensions were also not incorporated; therefore, Experiment 2 should be interpreted as assessing anticipated passage preference for socially structured visual apertures rather than actual body-scaled locomotor behavior. Although stimulus size was approximately standardized using the credit-card-based calibration procedure, screen resolution, viewing distance, and visual angle could not be strictly controlled. Finally, because participants could not respond during the 5-s stimulus presentation, the RT measure was not suited to examining the early temporal dynamics of responses to the stimuli.

Second, several aspects of the experimental design constrain interpretation and generalizability. Demographic information, including age, gender, and handedness, was not collected, preventing examination of potential individual differences. The study also lacked a non-social control condition, making it difficult to determine whether the observed effects were specific to social information per se. In addition, ΔDoP and CAD were not orthogonally manipulated, limiting the ability to clearly separate their contributions, and the study used a fixed set of eight avatar configurations without multiple independent exemplars. Future studies should therefore employ fully crossed manipulations, include non-social control conditions and multiple stimulus exemplars, and examine potential demographic influences.

Third, Experiment 1 used two physically identical apertures in a forced-choice task. Therefore, the observed effects demonstrate systematic biases in relative width judgments but do not directly establish that apparent aperture width was perceptually altered. Such responses could also reflect attentional or response biases. Psychophysical procedures that systematically vary physical aperture width and estimate the point of subjective equality would provide a stronger test of whether social configurations directly alter perceived width.

Future studies should extend the present findings using immersive VR or real-world locomotion paradigms that incorporate body-scaled information and dynamic interpersonal interactions. Such studies could examine whether the task-dependent CAD effect persists during actual movement and investigate mutual adaptation between pedestrians using eye-tracking and movement-trajectory analyses.

## 5 Conclusion

The present study demonstrated that the influence of social information on aperture choice can be task-dependent. While the relative social structure of the two apertures (ΔDoP) showed a similar association with choice across both perceptual and action-oriented tasks, with no evidence that its effect differed between tasks, the effect of the directional cue of the center avatar (CAD) differed significantly between tasks. Specifically, CAD showed opposite effects depending on whether participants were asked to compare spatial appearance or to anticipate passage. In addition, the descriptive RT–confidence association was stronger in Experiment 2 than in Experiment 1.

Taken together, these findings indicate that socially embedded spatial layouts do not exert fixed influences on behavior. Rather, their functional meaning is shaped by the constraints imposed by the task and by the way in which the perceiver is oriented toward potential action. In particular, directional information from the center avatar was associated with choice differently across task contexts.

Although the present findings were obtained in a controlled online setting, they provide initial evidence that even minimal directional cues within socially structured scenes can reorganize choice behavior when action is implied. These findings may also have practical implications for environmental design, as directional social cues may influence visual judgments and action-related choices differently depending on users’ intentions. Future research using immersive and movement-based paradigms will be essential to determine how these dynamics unfold in real-time navigation. In sum, the study highlights that socially structured space is not merely perceived but evaluated in relation to potential action: its influence depends on how individuals are positioned—both physically and functionally—within a field of potential movement.

## Data Availability

The raw data supporting the conclusions of this article will be made available by the authors, without undue reservation.
